# Individual variation in *Plasmodium vivax* malaria risk: Are repeatedly infected people just unlucky?

**DOI:** 10.1371/journal.pntd.0011020

**Published:** 2023-01-12

**Authors:** Rodrigo M. Corder, Ana Paula Arez, Marcelo U. Ferreira

**Affiliations:** 1 Department of Epidemiology and Biostatistics, University of California, Berkeley School of Public Health, Berkeley, California, United States of America; 2 Global Health and Tropical Medicine (GHTM), institute of Hygiene and Tropical Medicine, NOVA University of Lisbon, Lisbon, Portugal; 3 Department of Parasitology, Institute of Biomedical Sciences, University of São Paulo, São Paulo, Brazil; Universidade Federal de Minas Gerais, BRAZIL

## Abstract

Extensive research has examined why some people have frequent *Plasmodium falciparum* malaria episodes in sub-Saharan Africa while others remain free of disease most of the time. In contrast, malaria risk heterogeneity remains little studied in regions where *P*. *vivax* is the dominant species. Are repeatedly infected people in vivax malaria settings such as the Amazon just unlucky? Here, we briefly review evidence that human genetic polymorphism and acquired immunity after repeated exposure to parasites can modulate the risk of *P*. *vivax* infection and disease in predictable ways. One-fifth of the hosts account for 80% or more of the community-wide vivax malaria burden and contribute disproportionally to onward transmission, representing a priority target of more intensive interventions to achieve malaria elimination. Importantly, high-risk individuals eventually develop clinical immunity, even in areas with very low or residual malaria transmission, and may constitute a large but silent parasite reservoir.

## Introduction

Extensive research has examined why some people have repeated malaria episodes in Sub-Saharan Africa while others remain free of disease most of the time. To identify studies that describe malaria risk heterogeneity and their causes and consequences, especially in endemic settings where *Plasmodium vivax* is the dominant malaria-causing species, we searched the PubMed and SciELO databases for publications in English, Spanish, Portuguese, or French that appeared until Aug 1, 2021. We used the search terms (“risk heterogeneity” OR “superspreading” OR “Pareto” OR “overdispersed distribution” OR “genetic factors”) AND (“malaria” OR “*Plasmodium falciparum*” or “*Plasmodium vivax*). We also searched reference lists of identified studies.

## Why does individual malaria risk vary?

**Fig 1:**
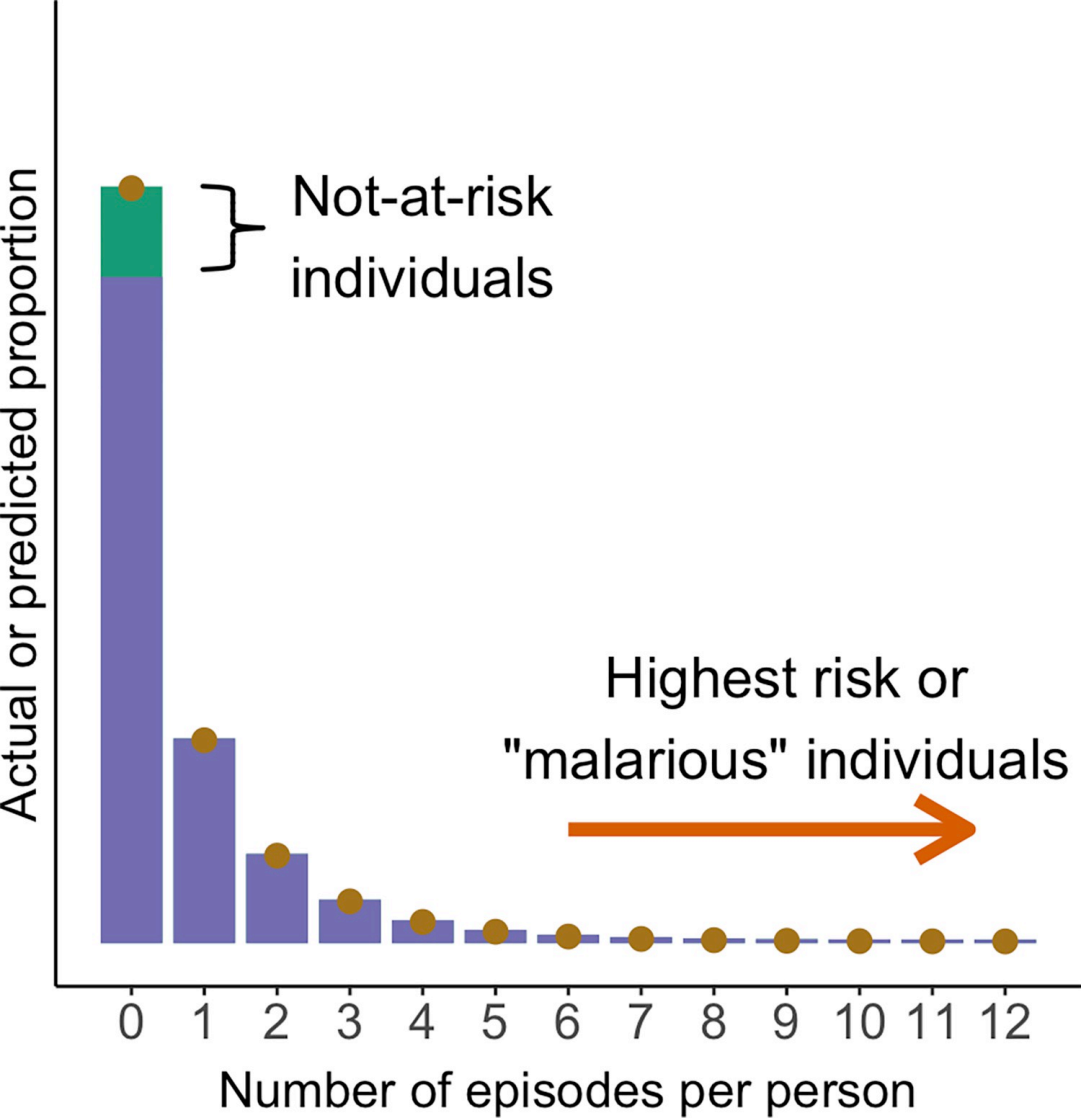
Zero-inflated negative binomial model (purple bars) fitted to overdispersed malaria episode counts per person (dots) over 33 months of follow-up in a population-based cohort in the Amazon Basin of Brazil. Counts of *P*. *falciparum* and *P*. *vivax* infections are combined (mean, 0.62 episodes; variance, 1.4). The not-at-risk (green bar segment) and highest-risk (orange arrow) fractions of the population are indicated. Redrawn from [2].

The burden of malaria is heterogeneously distributed in communities and tends to conform the 20/80 rule or Pareto principle: 20% of the individuals experience approximately 80% of the malaria episodes. Some malaria-exposed people have repeated malaria episodes, while most individuals remain free of disease over extended periods of time [1]. Fig 1 shows an example of the overdispersed frequency distribution of malaria episodes per person in the main transmission hotspot in the Amazon Basin of Brazil. A small fraction (<1%) of the population (“malarious people”) experienced 6 or more malaria episodes, while 67% had no malaria during the study [2].

The zero-inflated negative binomial (ZINB) fitted to incidence data combines the negative binomial distribution and the logistic model. It allows to simultaneously estimate the proportion of at-risk people, i.e., those who contribute cases following a negative distribution (including some individuals with zero counts—the “true zeroes”), and the proportion of people who are not at risk, the “structural zeroes” component described by the logistic model [3]. Not-at-risk people, estimated to comprise 14% of the population in Fig 1, remain malaria-free due to inherited resistance, limited exposure, or acquired immunity.

Why is malaria risk so heterogeneous and why are some people little susceptible or “protected”? To begin with, people differ in the frequency at which they are bitten by infected vectors [4]. Some individuals appear to be more attractive to mosquitoes than others [5], perhaps due to characteristics of their breath and sweat [6]. Pregnant women, for example, receive more bites from African malaria vectors than their nonpregnant counterparts from the same community or household [7]. Moreover, infected individuals may be more attractive to anopheline mosquitoes than their uninfected counterparts living in the same community [8]. Compared with children, adults are more likely to be bitten due to their greater body surface [9]. Some people may be more exposed to mosquito bites than others due to their behavior (e.g., time spent outdoors) or occupation (e.g., mining or farming in forest fringes), or due to contextual determinants (e.g., poor housing quality and living close to vector breeding sites).

Typically, 20% of the people experience approximately 80% of anopheline vector bites in high-endemicity settings [10–12]. Likewise, 20% of the exposed people are estimated to receive, on average, 80% of all *P*. *falciparum* sporozoite inoculations in typical rural African communities [11,13]. In areas with low endemicity in Africa, the top 1% individuals account for as many as 99% of the community-wide infectious mosquito bites [12].

Once sporozoites are inoculated by infected mosquitoes, not all individuals will develop blood-stage *P*. *falciparum* infection. Host genetics and immunity, among other factors, play a protective role. How much individual variation in malaria risk can be accounted for by genetic factors? Host genetics has been estimated to account for one-third to one-fourth of the variance in the risk of mild and severe *P*. *falciparum* malaria among children from coastal Kenya [14]. However, only a small proportion of the observed risk variance can be attributed to the most extensively studied genetic disorders that confer partial resistance to *P*. *falciparum* malaria, such as sickle-cell anemia and α-thalassemia, although these hemoglobinopathies are common in the local host population [14].

Adaptive immunity is another key factor that originates malaria risk heterogeneity. For example, some African children appear to develop immunity faster and tend to control parasite multiplication and prevent clinical malaria earlier than other children exposed to the same environment [15]. Recent research into clinical immunity in malaria has examined antigen-specific immune responses that appear to modulate the risk of infection, uncomplicated malaria or severe disease (e.g., [16]). However, the search for immune correlates of protection is confounded by several factors, including the marked individual variation in exposure to infection that blurs the distinction between functional immune responses and those that are acquired simply as a result of repeated infections, but are not necessarily protective [17].

Finally, infected individuals in Africa differ in their ability to transmit *P*. *falciparum* to mosquitoes. Some people are super-spreaders, as defined by Lloyd-Smith and colleagues [18]: They consistently carry infective gametocytes for longer periods of time, are more often bitten by mosquitoes, or a combination of both [12]. Model simulations indicate that malaria control could be substantially more effective if targeted to super-spreaders, as long as they can be readily identified in communities [13].

## Relapses and repeated *Plasmodium vivax* infections

Variation in individual risk of malaria has been little studied in areas where *P*. *vivax* is the dominant parasite, such as the Amazon, where the distinctive parasite’s biology poses additional challenges for malaria elimination [19,20]. Nearly 3.3 billion people are at risk of infection with *P*. *vivax* and 14.3 million cases are estimated to occur yearly [21].

We hypothesize that several factors can modulate the individual risk of *P*. *vivax* infection and disease in a species-specific manner. For example, factors that affect *P*. *vivax* hypnozoite survival and activation can promote or prevent relapses [22]. Although there is no direct evidence for primaquine (PQ)-resistant hypnozoites [23], nearly 11% of *P*. *vivax* infections are estimated to relapse within 12 months in the Amazon despite routine PQ prescription [24]. Not surprisingly, relatively few *P vivax*-infected people typically contribute the vast majority of relapses [25]. Therefore, some people are repeatedly infected with *P*. *vivax* simply because they are more prone to relapses than others, e.g., due to factors that preclude PQ administration or reduce its efficacy.

First, some patients cannot take PQ. Patients with severe forms of glucose-6-phosphate dehydrogenase (G6PD) deficiency, a common hereditary disorder in malaria-endemic settings [26], may develop life-threatening hemolysis following treatment [27]. Moreover, PQ is contraindicated for pregnant women and children below 6 months of age, because of the risk of hemolysis in fetuses and infants of unknown G6PD status [27]. Finally, not all patients adhere to the current multiple-dose PQ regimens used in the radical cure of vivax malaria [28,29]. Seven-day PQ regimens may improve patient adherence, but there is no evidence that they are more effective in preventing relapses compared to the usual 14-day regimen [30].

Second, some patients cannot properly metabolize PQ, which is an inactive prodrug. Because biotransformation mediated by the cytochrome P450 (CYP) isoenzyme CYP2D6 is required for PQ antirelapse activity [31], patients carrying low-activity CYP2D6 variants may relapse despite taking PQ [32]. There is extensive genetic diversity at the *cyp2d6* locus, with over 130 alleles that are associated with loss of activity, decreased, normal, or increased function (https://www.pharmvar.org/gene/CYP2D6). Four phenotypes can be inferred from genotypes: (i) poor metabolizer (two nonfunctional alleles); (ii) intermediate metabolizer (one normal and one functionally deficient allele); (iii) normal metabolizer (two functional alleles); and (iv) ultrarapid metabolizer (at least one increased function allele—i.e., multiple copies of a functional allele on one chromosome—in addition to a functional allele) [33].

Low-activity CYP2D6 variants are commonly seen in populations exposed to *P*. *vivax* transmission. For example, poor and intermediate metabolizers account for 20% to 35% of vivax malaria patients in the Amazon Basin of Brazil [24–36]. Carriers of low-activity CYP2D6 alleles were found to be at increased risk of *P*. *vivax* recurrence following chloroquine-PQ treatment in some [32,34,36], although not all, clinical studies [35,37]. Importantly, high-dose PQ treatment may represent a safe and efficacious alternative to circumvent poor or intermediate CYP2D6-mediated metabolization in Brazil [37]. The impact of CYP2D6 polymorphism on community-wide malaria risk variation remains undetermined, despite the high prevalence of low-activity variants in some endemic settings.

## Human genetics, red blood cell invasion, and *P*. *vivax* malaria risk variation

The susceptibility to blood-stage *P*. *vivax* infection is likely to be modulated by several inherited characteristics of the hosts—including a wide range of genes that regulate innate and adaptive immune responses [38]. Host genetics may either favor or block red blood cell (RBC) invasion and the intracellular multiplication of parasites [19,39], but the relative contribution of genetic versus nongenetic factors (e.g., sociodemographic, environmental) to individual variation in *P*. *vivax* malaria risk has not been estimated at the population level.

Asexual blood stages of *P*. *vivax* display two unique biological characteristics: (i) merozoites can only invade immature RBCs, known as reticulocytes; and (ii) merozoites must typically interact with the Duffy antigen receptor for chemokines (DARC) to invade RBC. Reticulocytes account for only 0.5% to 1.5% of circulating RBCs in healthy adults, but health conditions that accelerate the RBC turnover rate, such as inherited hemolytic anemias, increase the proportion of young RBCs in the peripheral blood that can be infected by *P*. *vivax*. Hemoglobinopathies such as sickle-cell anemia and α-thalassemia are examples of conditions associated with chronic reticulocytosis, but whether they favor *P*. *vivax* infection by increasing the number of suitable host cells in the peripheral blood remains largely undetermined [40]. α-thalassemia protects African children from severe *P*. *falciparum* malaria but may increase the risk of uncomplicated *P*. *vivax* infection in some [41], although not all, Melanesian children [42]. Likewise, pyruvate kinase deficiency, a hereditary RBC enzyme defect that causes nonspherocytic hemolytic anemia, limits *P*. *falciparum* growth in human erythrocytes [43,44] but may increase the risk of vivax malaria [45].

The interaction between DARC and the *P*. *vivax* adhesin Duffy binding protein (PvDBP) is critical for RBC infection [19]. Importantly, DARC polymorphism is a key determinant of susceptibility to *P*. *vivax* infection and disease, with no effect on *P*. *falciparum* risk—as the latter species explores other receptors to invade RBCs. The main polymorphism that reduces the susceptibility to *P*. *vivax* infection is a T→C nucleotide substitution in the erythrocyte-specific GATA1 transcription factor–binding motif, which abolishes DARC expression on RBCs [46]. This leads to the Duffy-negative phenotype, which is widespread in human populations from West and Central Africa and causes blood-stage *P*. *vivax* infections to be infrequent in this region [47]. Since the mid-1970s, Duffy-negative individuals are known to be refractory to blood-stage *P*. *vivax* infection [48], but the parasite appears to have partially overcome this invasion blockade in parts of Africa and the Amazon [47], possibly by using alternate RBC receptors [49] or by increasing the number of copies of PvDBP [50–52]. A second common nucleotide substitution, A→G in codon 42 of the DARC gene, defines the Fy^a^ antigen, which causes reduced PvDBP binding and may lower the risk of *P*. *vivax* infection [53].

In addition to hemoglobinopathies, other inherited hemolytic anemias that protect against *P*. *falciparum* infection and/or severe disease may also affect susceptibility to *P*. *vivax* [54]. One example is Southeast Asian ovalocytosis, an intrinsic RBC membrane defect caused by a 27 base-pair deletion of band 3 that is lethal in the homozygous state. Heterozygosity is very common in some coastal populations of Papua New Guinea and confers strong protection against *P*. *vivax* malaria [55].

G6PD deficiency, the most common RBC enzymopathy, also affects malaria risk. The relatively mild West African G6PD A− variant (10% to 60% G6PD activity), found in 39.3% of the G6PD-deficient Amazonians [56], is associated with a significant reduction in the risk of severe falciparum malaria in both male hemizygotes and heterozygous females in Africa. Whether the G6PD A− variant affects the risk of *P*. *vivax* infection and disease is unknown [47]. Nevertheless, the Mediterranean variant (1% to 10% G6PD activity), which is widely distributed across Europe and Asia [26] but rare in the Amazon [56], reduces the risk of symptomatic *P*. *vivax* malaria by 76% in hemizygous males and homozygous females of Pashtum ethnicity from Afghanistan [57,58].

## Variation in peripheral *P*. *vivax* density and its consequences

Once infected with *P*. *vivax*, few people present relatively high peripheral blood parasite densities because of the strict reticulocyte tropism, while most maintain low-grade infections. A number of factors that affect the parasite’s ability to invade and multiply within RBCs—e.g., reticulocyte availability in the peripheral blood and hematopoietic niches, RBC polymorphism, intrinsic parasite multiplication rate, superinfection, and innate and adaptive immunity—will determine the overall parasite biomass harbored by each host.

**Fig 2:**
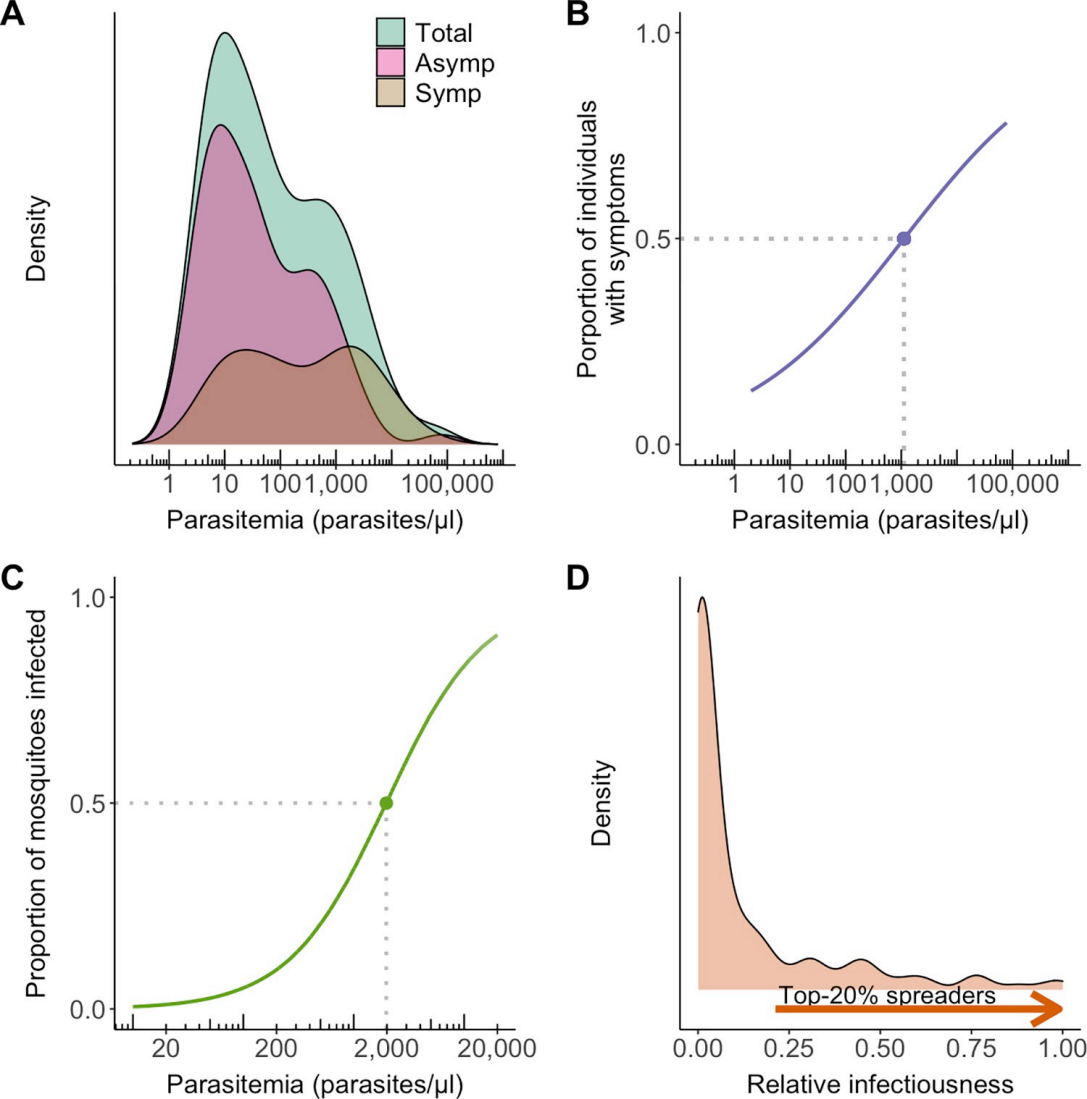
How blood-stage parasite density relates to the risk of clinical manifestations and human-to-mosquito transmission of *Plasmodium vivax*. (**A**) Distribution of parasite densities measured by quantitative PCR in symptomatic (“Symp”) and asymptomatic (“Asymp”) *P*. *vivax* infections. (**B**) Association between *P*. *vivax* blood-stage density and the risk of clinical symptoms (fever or headache) within 7 days prior to detection of blood-stage infection, with >50% of the individuals being symptomatic above 1,100 parasites/μL. (**C**) Association between total *P*. *vivax* blood-stage density and the probability of *Anopheles darlingi* infection in standard membrane feeding assays, with 50% of the mosquitoes being infected at approximately 2,000 parasites/μL, assuming that 15% of circulating parasites are mature gametocytes. (**D**) Relative infectiousness of infected individuals in the community. We combine individual estimates of relative infectiousness of *P*. *vivax* carriers in the community. Most infected people are little infectious (relative infectiousness close to zero), but the top 20% spreaders are estimated to account for nearly 80% of all transmission events. Data from a farming settlement in the Amazon Basin of Brazil [63]. Figures redrawn from [59].

At the community level, *P*. *vivax* densities in the bloodstream are widely variable both in symptomatic and asymptomatic people (Fig 2A) [59,60]. Such variation has clear clinical implications, as the risk of clinical disease relates directly to peripheral blood *P*. *vivax* density. Simply put, people with high parasitemia are more likely to have clinical manifestations and complications. Clinical or “fever” thresholds in *P*. *vivax* infection vary widely among populations, but most individuals in the Amazon are expected to develop malaria-related symptoms at densities above 200 to 1,000 parasites per microliter of blood (Fig 2B). Clinical thresholds are one order of magnitude higher in children than in adults living in the same communities [59]—as previously found in *P*. *falciparum* infections in Africa [61]. Importantly, not all parasites circulate in the bloodstream; *P*. *vivax*-infected RBCs tend to accumulate in hematopoietic tissues such as the bone marrow, where reticulocytes are more abundant, and in the spleen [62]. As a result, peripheral blood parasite density may not predict accurately the total parasite burden in *P*. *vivax* infections.

High-density infections also have important public health consequences. Although mature gametocytes can be detected by molecular methods in nearly all *P*. *vivax* blood-stage infections [63], membrane feeding assay data show that not all human hosts are equally infectious to local vectors [64,65]. Indeed, *P*. *vivax* infectiousness to mosquitoes correlates positively with the number of gametocytes circulating in the peripheral blood [59,66]. For example, very few Amazonian vectors can be experimentally infected at the low *P*. *vivax* gametocyte densities typically found in most local hosts, but the infection rate reaches 50% at 340 gametocytes/μL, which corresponds to a total parasitemia of approximately 2,300/μL [59] (Fig 2C). Under the simplifying assumption that host’s infectiousness depends primarily on their age, which correlates positively with the number of mosquito bites received [9], and on their peripheral parasite densities (Fig 2C), we estimate that most infected individuals are very little infectious (Fig 2D). Conversely, the top 20% spreaders (or “super-spreaders”) originate between 79% and 93% of all *P*. *vivax* transmission events to mosquitoes in Amazonian communities [59]—offering a further example of the 20/80 rule in malaria epidemiology.

## Asymptomatic *P*. *vivax* reservoir in low-endemicity settings

A vast, clinically silent human reservoir of *P*. *vivax* infection persists in areas approaching malaria elimination across the Americas and challenges the long-standing dogma that infections necessarily lead to overt disease in populations exposed to low-level transmission, due to the lack of acquired clinical immunity [67]. How residual, low-level malaria transmission outside Africa reconciles with the development of clinical immunity to *P*. *vivax* remains uncertain, but malaria risk heterogeneity may provide some clues [68].

The individual risk of vivax malaria varies widely at the community level as a result of sociodemographic, genetic, and behavioral factors. The paradoxical finding of asymptomatic infections in low-endemicity settings might be explained by the presence of a minority of high-risk people who are repeatedly exposed to blood-stage parasites and eventually become immune, while low-risk individuals are seldom infected and remain susceptible to infection and disease during their lifetime. To estimate the proportion of high-risk individuals living in Brazil’s main malaria hotspot, we fitted compartmental susceptible-infected-susceptible (SIS)-type transmission models simultaneously to (i) age-stratified vivax malaria incidence densities and (ii) the frequency distribution of *P*. *vivax* malaria episodes experienced by each individual over time [68]. Instead of assuming that all individuals are uniformly susceptible to infection and disease, we discretized individual risk in two groups *j* (*j* = 1 for low risk [LR] and *j* = 2 for high risk [HR]) to describe the transmission dynamics of *P*. *vivax* malaria [68]. Each risk group comprises a proportion *p*_*j*_ (0<*p*_*j*_<1; *j* = 1; 2 and *p*_1_+*p*_2_ = 1) of the population and is associated with a risk factor *x*_*j*_>0 (*j* = 1; 2). We assume that the overall average risk is equal to 1 (*x*_1_*p*_1_+*x*_2_*p*_2_ = 1), since the factors *x*_*j*_ are modifiers of individual responses to a force of infection that was allowed to vary. This setting configures a risk distribution with variance *v* = *p*_1_(*x*_1_−1)^2^+*p*_2_(*x*_2_−1)^2^.

**Fig 3:**
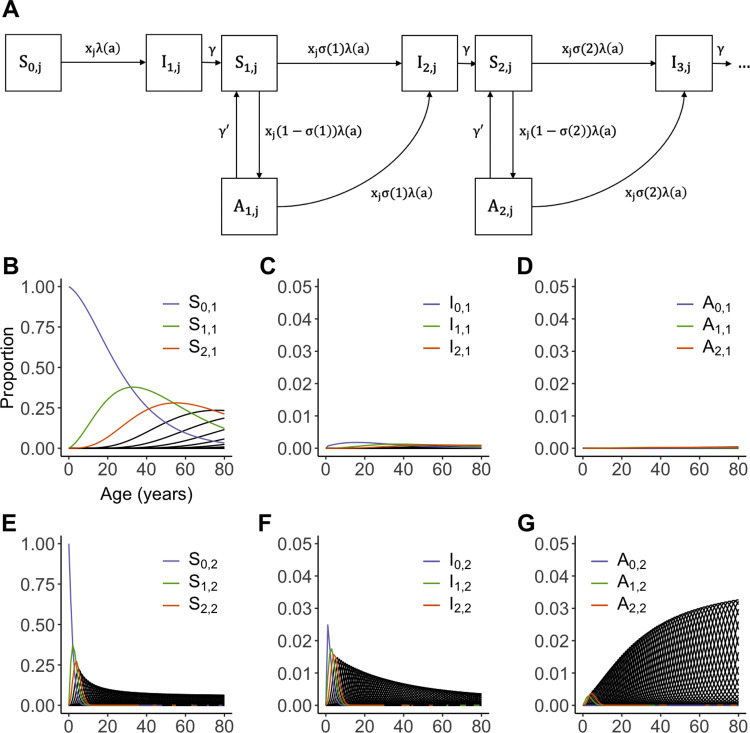
Transmission dynamics of *Plasmodium vivax* in a heterogeneous population. (**A**) SIS-type model used to describe the dynamics of symptomatic and asymptomatic *Plasmodium vivax* infections across age groups in LR and HR individuals. Compartment *S*_*i*,*j*_ represents susceptible but uninfected individuals from risk group *j* who have experienced *i* prior clinical malaria. At birth, individuals are allocated to compartment *S*_0,*j*_. Compartment *I*_*i*,*j*_ represents individuals from risk group *j* currently experiencing their *i*^*th*^ clinical vivax malaria episode Finally, compartment *A*_*i*,*j*_ represents individuals from risk group *j* with *i* past clinical malaria episodes who are currently experiencing an asymptomatic *P*. *vivax* infection. The next panels show the dynamics of the following: (**B**) Susceptible but uninfected LR individuals; (**C**) Infected and symptomatic LR individuals; (**D**) Infected and asymptomatic LR individuals; (**E**) Susceptible but uninfected HR individuals; (**F**) Infected and symptomatic HR individuals; and (**F**) Infected and asymptomatic HR individuals. Redrawn from [68]. HR, high-risk; LR, low-risk; SIS, susceptible-infected-susceptible.

Assuming equilibrium, in our modeling approach (represented diagrammatically in Fig 3A), malaria unfolds in the age domain according to an age(*a*)-dependent force of infection *λ*(*a*) [69] modified by risk factors *x*_*j*_, according to the risk group *j*, a partial immunity weight *σ*(*i*) = *e*^−*α*∙*i*^ (which *i* being the number of malaria episodes) and the recovery rates *γ* and *γ*′ of infected symptomatic and asymptomatic individuals, respectively. For each risk group *j*, we obtained the dynamics of individuals according to their epidemiological status: susceptible (compartments *S*_*i*,*j*_; Fig 3B and 3E), infected and symptomatic (compartments *I*_*i*,*j*_; Fig 3C and 3F), or infected and asymptomatic (compartment *A*_*i*,*j*_; Fig 3D and 3G). Importantly, although we do not consider relapses explicitly in our model approach, they are implicitly integrated into the force of infection, which combines blood-stage infections arising from infecting stages (sporozoites) inoculated during mosquito bites and relapses arising from reactivating hypnozoites. The best-fitting model suggests that 20% of the population is under high-risk and, assuming that asymptomatic infections last 90 days on average (i.e., *γ*′. = 1/90 per day), this group is estimated to contribute 86% of the overall vivax malaria burden. The model also suggests that as many as 25 symptomatic *P*. *vivax* infections per individual, on average, are required to reduce the probability of developing clinical malaria risk by half [68]. Some people essentially from the HR group experience enough infections during their childhood and adolescence to acquire clinical immunity as young adults and eventually constitute the asymptomatic parasite reservoir. Individuals assigned to the low-risk group contribute very little to the overall burden of infection and disease.

In addition to acquired immunity, differences in parasite virulence might also account for the high proportion of asymptomatic infections at times of low transmission [70]. Virulent parasites—those with high multiplication rates, which more commonly cause disease—are hypothesized to have a selective advantage in high-transmission settings, where repeated infections and superinfection events are common. Virulent parasites are more likely to survive the fierce within-host competition for limited resources between coinfecting clones and species, as often observed in experimental rodent malaria models (e.g., [71]). In contrast, nonvirulent parasites may have a selective advantage in low-transmission settings, where hosts are more likely to harbor single-species and single-clone asymptomatic infections that remain undiagnosed and untreated, and may contribute to transmission over several weeks or months [70]. The “virulence hypothesis” may similarly apply to sharply seasonal *P*. *falciparum* transmission [72] as well as to declining *P*. *vivax* transmission elsewhere in the tropics [73].

## Concluding remarks

Repeatedly infected people—including relapse-prone individuals who cannot take or metabolize PQ—are commonly found even in communities with relatively low *P*. *vivax* transmission [2], with important clinical consequences. Unsurprisingly, repeated *P*. *vivax* infections are more likely to cause adverse clinical outcomes—e.g., anemia among PQ-ineligible pregnant women and infants [74–76].

Risk heterogeneity has also clear implications for malaria control and elimination. The 20/80 rule implies that control programs selectively targeted at high-risk people are more likely to be effective [10,13]. Indeed, mathematical model simulations suggest that imperfect control measures, such as leaky vaccines, if uniformly applied to all hosts, are unlikely to reduce substantially the malaria burden in a population with widely variable risk of infection [77]. The next challenge is to identify “malarious people”—individuals who contribute most to *P*. *vivax* infection and transmission in the population—to be targeted to more intensive and effective measures.

**Key Learning Points**

- The burden of *Plasmodium vivax* malaria in communities follows the 20/80 rule or Pareto principle: 20% of the people account for 80% or more of all infections.
- Factors that preclude the use of primaquine to eliminate hypnozoites (e.g., pregnancy) or reduce its efficacy (e.g., low activity of the cytochrome P450 isoenzyme CYP2D6) may specifically increase the burden of *P*. *vivax* disease and transmission potential by rendering some people more likely to have relapses.
- Inherited hemolytic anemias, caused by defects in hemoglobin synthesis, RBC membrane structure, or RBC enzymes, are classically regarded as “malaria resistance” factors but do not necessarily lower the risk of vivax malaria.
- Human polymorphisms known to reduce the susceptibility to *P*. *vivax* infection (e.g., Duffy blood group negativity) or affect the efficacy of antirelapse treatment (e.g., low-activity CYP2D6 variants) have no such effects on falciparum malaria risk; these are “vivax-specific” malaria resistance factors.
- Mathematical modeling must consider individual risk variation to describe the transmission dynamics of *P*. *vivax* and the impact of control interventions, especially those selectively targeted at high-risk individuals.

**Top Five Papers**

1. Baird JK, Battle KE, Howes RE. Primaquine ineligibility in anti-relapse therapy of *Plasmodium vivax* malaria: the problem of G6PD deficiency and cytochrome P-450 2D6 polymorphisms. Malar J. 2018;17:42. doi: 10.1186/s12936-018-2190-z
2. Corder RM, Ferreira MU, Gomes MGM. Modelling the epidemiology of residual *Plasmodium vivax* malaria in a heterogeneous host population: a case study in the Amazon Basin. PLoS Comput Biol. 2020;16:e1007377. doi: 10.1371/journal.pcbi.1007377
3. Ferreira MU, Corder RM, Johansen IC, Kattenberg JH, Moreno M, Rosas-Aguirre A, et al. Relative contribution of low-density and asymptomatic infections to *Plasmodium vivax* transmission in the Amazon: pooled analysis of individual participant data from population-based cross-sectional surveys. Lancet Reg Health Am. 2022;9:100169. doi: 10.1016/j.lana.2021.100169
4. Taylor SM, Fairhurst RM. Malaria parasites and red cell variants: when a house is not a home. Curr Opin Hematol. 2014;21:193–200. doi: 10.1097/MOH.0000000000000039
5. Woolhouse ME, Dye C, Etard JF, Smith T, Charlwood JD, Garnett GP, et al. Heterogeneities in the transmission of infectious agents: implications for the design of control programs. Proc Natl Acad Sci U S A. 1997;94:338–42. doi: 10.1073/pnas.94.1.338
